# Senescence Is Associated With Elevated Intracellular Resting [Ca^2 +^] in Mice Skeletal Muscle Fibers. An *in vivo* Study

**DOI:** 10.3389/fphys.2020.601189

**Published:** 2021-01-12

**Authors:** Alfredo Mijares, Paul D. Allen, Jose R. Lopez

**Affiliations:** ^1^Centro de Biofísica y Bioquímica, Instituto Venezolano de Investigaciones Científicas, Caracas, Venezuela; ^2^Malignant Hyperthermia Investigation Unit, St James’ University Hospital, University of Leeds, Leeds, United Kingdom; ^3^Department of Research, Mount Sinai Medical Center, Miami, FL, United states

**Keywords:** aging, calcium, TRPC, skeletal muscle, inflammation

## Abstract

Aging causes skeletal muscles to become atrophied, weak, and easily fatigued. Here, we have tested the hypothesis that normal aging in skeletal muscle cells is associated with Ca^2+^ intracellular dyshomeostasis and oxidative stress. Intracellular Ca^2+^ concentration ([Ca^2+^]_i_), resting intracellular Na^+^ concentration ([Na^+^]_i_) and reactive oxygen species (ROS) production were measured *in vivo* (superficial gastrocnemius fibers) using double-barreled ion-selective microelectrodes, and *in vitro* [isolated single flexor digitorum brevis fibers] using fluorescent ROS sensor CM-H2DCFDA in young (3 months of age), middle-aged (12 months of age), and aged (24 months of age) mice. We found an age-related increase in [Ca^2+^]_i_ from 121 ± 4 nM in young muscle cells which rose to 255 ± 36 nM in middle-aged and to 409 ± 25 nM in aged cells. [Na^+^]_i_ also showed an age-dependent elevation, increasing from 8 ± 0.5 mM in young muscle fibers, to 12 ± 1 mM in middle-aged and to 17 ± 1 mM in old muscle fibers. Using the fluorescent ROS sensor CM-H2DCFDA we found that these increases in intracellular cation concentrations were associated with significantly increased basal ROS production as demonstrated by age related increases in the rate of dichlorodihydrofluorescein fluorescence. To determine is this could be modified by reducing ROS and/or blocking sarcolemmal Ca^2+^ influx we administered flufenamic acid (FFA), a non-steroidal anti-inflammatory drug which is also a non-selective blocker of the transient receptor potential canonical channels (TRPCs), for 4 weeks to determine if this would have a beneficial effect. FFA treatment reduced both basal ROS production and muscle [Ca^2+^]_i_ and [Na^+^]_i_ in middle-aged and aged muscle fibers compared to fibers and muscles of untreated 12 and 24-months old mice. [Ca^2+^]_i_ was reduced to 134 ± 8 nM in middle-aged muscle and to 246 ± 40 nM in muscle from aged mice. Likewise [Na^+^]_i_ was reduced to 9 ± 0.7 mM in middle-aged muscles and to 13 ± 1 mM in muscle from aged mice. FFA treatment also reduced age associated increases in plasma interleukin 6 and tumor necrosis factor-alpha (TNF-α) concentrations which were elevated in 12 and 24-months old mice compared to young mice and decreased age-related muscle damage as indicated by a reduction in serum creatine kinase (CK) activity. Our data provides a direct demonstration that normal aging is associated with a significant elevation [Ca^2+^]_i_, [Na^+^]_i_, and intracellular ROS production in skeletal muscle fibers. Furthermore, the fact that FFA reduced the intracellular [Ca^2+^], [Na^+^], and ROS production as well as the elevated IL6, TNF-α, and CK levels, led us to suggest that its pharmacological effect may be related to its action both as a TRPC channel blocker and as an anti-inflammatory.

## Introduction

Aging is associated with a concomitant reduction in skeletal mass and muscle strength with a lack of causal disease (Rolland et al., 2008; Tieland et al., 2018). The etiology of muscle aging is complex and still is not fully elucidated. Diverse alterations in muscle have been described, e.g., a significant decrease in myofiber size and number, improper protein synthesis–degradation, and a reduction in the magnitude of motor neurons innervating muscle cells (Doherty, 2003; Mosole et al., 2014). The reduction in muscle mass and strength observed during senescence compromises physical activity promoting a sedentary lifestyle that predisposes the individual to the risk of falling and even death (Visser and Schaap, 2011). Dysfunctional excitation–contraction coupling (Delbono et al., 1995), reduced density of calcium release units (Boncompagni et al., 2006), increased oxidative stress, and alterations of mitochondrial function appears to play a role in age-related changes in muscle (Pietrangelo et al., 2015).

Although abnormalities in intracellular calcium regulatory mechanisms in skeletal muscle with aging have been suggested, no systematic study has been conducted. To our knowledge, the present study represents the first attempt to determine the impact of senescence on intracellular Ca^2+^ concentration ([Ca^2+^]_i_) in skeletal muscle, and the role of Ca^2+^ influx mediated by the transient receptor potential canonical (TRPC channels) observed with aging. In addition, we also probed the contribution of oxidative stress and the inflammatory cytokines interleukin (IL-6) and tumor necrosis factor-alpha (TNF-α). We hypothesized that senescence is associated with a chronic increase in [Ca^2+^]_i_ and resting intracellular Na^+^ ([Na^+^]_i_) in skeletal muscle cells that could be modified or normalized with pharmacological intervention with flufenamic acid (FFA), which is both a non-steroidal anti-inflammatory (NSAID) drug and a non-selective blocker of TRPC channels (Foster et al., 2009; Suzuki et al., 2011; Jiang et al., 2012). We found that treatment with FFA reduced [Ca^2+^]_i_ and [Na^+^]_i_, decreased production of reactive oxygen species (ROS), lowered cytokine release, and diminished muscle damage as indicated by a reduction of plasma creatine kinase (CK) activity.

## Materials and Methods

### Animals and Experimental Groups

Young – 3 months; middle-aged – 12 months, and aged – 24 months old C57BL/6J male mice were maintained in the Mount Sinai Medical Center, United States vivarium under constant temperature (21–22°C) on a 12-h light/12-h dark cycle with free access to food and water. All experimental procedures were approved by the Institutional Animal Care and Use Committee at Mount Sinai Hospital.

The first cohort of mice was used for *in vivo* intracellular ion measurements in muscle and consisted of five groups of mice: (A) Control group: Mice (*N* = 6) that did not receive any treatment for the 24 months study and had *in vivo* intracellular Ca^2+^ and Na^+^ determinations performed serially at 3, 12, and 24 months. (B) Experimental group 1: 8-week old mice (*N* = 7) were treated for 4 weeks with flufenamic acid (FFA-an anti-inflammatory and non-selective blocker of TRPC channels) injected intraperitoneally (IP) (12.5 mg/kg) once per day for 4 weeks and *in vivo* intracellular Ca^2+^ and Na^+^ determinations were carried out at the age of 3-months. (C) Experimental group 2: 44-week old mice (*N* = 6) were treated with FFA for 4 weeks, and *in vivo* intracellular Ca^2+^ and Na^+^ determinations were carried out at the age of 12-months. (D) Experimental group 3: 92-week-old mice (*N* = 5) were treated with FFA for 4 weeks, and *in vivo* intracellular Ca^2+^ and Na^+^ determinations were carried out at the age of 24-months.

The second cohort of 24 mice were randomly divided into six groups (three treatment and three control, *N* = 4 mice per group) and given the same treatment that was used in the first cohort at 8, 44, and 92 weeks and the studies carried out at 3, 12, and 24 months of age. In this cohort, enzymatically dissociated isolated single flexor digitorum brevis (FDB) muscle cells were used to determine ROS production rate using a fluorescence assay. Unlike the first group, where it was possible to make serial measurements in the control group, separate control groups were used for each time point.

The third cohort of 36 mice of each age were randomly divided into six groups (three treatment and three control*s, N* = 6 mice per group) and given the same treatment given as in the first cohort at 8, 44, and 92 weeks and the studies carried out at 3-, 12- and 24-months of age. At that time, the mice blood was collected from the tail vein for measurements of plasma IL-6, TNF-α, and CK concentrations, and the animals were then euthanized by cervical dislocation.

### Ca^2+^- and Na^+^-ion Selective Microelectrodes

Double-barreled, Ca^2+^- or Na^+^-selective microelectrodes were prepared from thin-walled 1.2- and 1.5-mm outside diameter (OD) borosilicate HCl-washed glass capillaries (PB150F-4, World Precision Instruments, FL, United States). They were heat sterilized (UX-10776-00, Cole Palmer, IL, United States) stretched in a pipette puller (P-97 Flaming/Brown, Automate Scientific, Berkeley, CA, United States) to obtain small-tipped microelectrodes (≤1 μM). The ions-selective barrel (1.5-mm OD) was silanized by exposing it to dimethyldichlorosilane vapor. The tip was then back-filled with either the Ca^2+^ ionophore II (ETH 129, Sigma-Aldrich, MO, United States) or Na^+^ ionophore (ETH 227, Sigma-Aldrich, MO, United States). The barrel’s remainder was back-filled with pCa7 for the Ca^2+^ selective microelectrode or 8 mM NaCl solution for the Na^+^ selective as described previously (Eltit et al., 2013). The membrane potential barrel (1.2-mm OD) was back-filled with 3 M KCl just before the measurements were performed. The tip resistances were measured by passing a current pulse of 1 pA through an individual barrel while the electrode tip was in the Ringer bathing solution (Eltit et al., 2013). For the Ca^2+^ and Na^+^-selective microelectrodes, the resistances ranged from 8 to 10 × 10^10^ Ω, and for the membrane potential microelectrodes (Vm), the resistances ranged from 10 to 15 MΩ with a tip potential less than 5 mV. The double-barreled ion-selective microelectrode was mounted in a modified plastic holder containing Ag/AgCl wires. It was attached to a miniature head stage (probe input impedance > 10^11^Ω), which was connected to a Duo 773 electrometer (World Precision Instruments, FL, United States). The Vm and ions specific potentials were acquired at a frequency of 1,000 Hz with AxoGraph software (version 4.6; Axon Instruments, CA, United States) and stored in a computer for further analysis. Each ion-selective microelectrode was individually calibrated before and after the measurement by exposure of the tip to a series of calibrating solutions, as described previously, and if the two calibration curves did not agree within 3 mV, data from that microelectrode were discarded (Lopez et al., 1983; Eltit et al., 2013). The bath’s reference electrode was either an Ag–AgCl pellet or an agar bridge made of a polythene tube containing 3 M KCl gelled in agar. Muscle cells were impaled with the double-barreled ion-microelectrode and Vm and Ca^2+^ or Vm and Na^+^ potentials were measured. After both potentials were stable for at least 1 min, the microelectrode was withdrawn.

### Recording of Intracellular [Ca^2+^] and [Na^+^] in Muscle Fibers *in vivo*

Intracellular Ca^2+^ and Na^+^ determinations were carried out *in vivo* using Ca^2+^- and Na^+^-selective microelectrodes (Eltit et al., 2013) under aseptic conditions. After the mice were anesthetized, the hair on the leg was removed, the skin was cleaned with an antiseptic solution, and a small incision was made. The gastrocnemius muscle was identified, the muscle fascia was partially removed, and the superficial fibers were exposed. Warm sterile Ringer’s solution was perfused onto the superficial muscle fibers to preserve moisture. The mice were kept euthermic (37°C) with the aid of a low noise heating system (ATC1000, World Precision Instruments, FL, United States). Through the aid of a stereomicroscope (233445 Olympus, MA, United States), individual muscle fibers were impaled with either Ca^2+^ or Na^+^ double-barreled microelectrodes. Examples of actual recordings from these electrodes can be found in our previous publications (Lopez et al., 1983, 2000, 2011, 2020).

In the control group, [Ca^2+^]_i_ and [Na^+^]_i_ measurements were repeated in the same animal at all three-time points (3, 12, and 24-months). In this group, after the measurements of [Ca^2+^]_i_ and [Na^+^]_i_ were made, the area around the wound was washed with streptomycin solution (10 mg/L), and the skin was sutured. Topical local anesthetic (bupivacaine) was applied every 12 h for 48 h to the wound area to relieve minor pain (type A – wound suturing) produced by survival surgery according to the Guide for the Care and Use of Laboratory Animals. In the treated groups and the control group at 24 months, after completing measurements of [Ca^2+^]_i_ and [Na^+^]_i_, mice were euthanized by cervical dislocation. Before making measurements, all mice were kept in individual cages to avoid potential damage by other mice.

### Determination of Reactive Oxygen Species in Muscle Cells

Intracellular ROS levels were determined in enzymatically isolated FDB muscle cells from treated and untreated 3, 12, and 24 month-old mice using the dichlorodihydrofluorescein diacetate (DCFHDA) assay (Luis, MO, United States) as previously described (Lopez et al., 2018b). The fluorescence intensity of dichlorodihydrofluorescein (DCF) was detected by a fluorescence microplate reader (Molecular Device, Sunnyvale, CA, United States) at an excitation wavelength of 488 nm and an emission wavelength 525 nm. All measurements were performed in triplicate, and the results reported as the percentage of ROS production relative to untreated 3-month muscle cells.

### Determination of Plasma IL6 and TNF-α Concentrations and CK Activity

Plasma IL-6 concentrations were measured in plasma samples from blood collected from the tail vein in treated and untreated 3, 12, and 24 months-old animals using a Milliplex Mouse Cytokine/Chemokine Panel (EMD Millipore, MA, United States) with a Bio-Plex Suspension Array System (Bio-Rad Laboratories, CA, United States). Similarly, TNF-α content was measured in plasma samples from untreated and treated 3, 12, and 24 months-old animals using a mouse TNF-α ELISA kit (Invitrogen, CA, United States). The values were normalized to muscle protein concentration determined by BCA protein assay (Thermo Scientific, MA, United States). Plasma CK activity was determined using a creatine kinase assay kit (Sekisui Diagnostics, MA, United States) according to manufacturer’s instructions. CK activity was determined in triplicate from each sample and expressed as units per liter (U/L). All measurements were carried out on triplicate blood samples obtained from the same animal at three different times on the same day (8 am, 12 pm, and 4 pm). Despite the small blood volume withdrawn, oral fluid replacement was provided between each endpoint.

### Statistics

Data are reported as mean ± standard deviation (SD). For [Ca^2+^]_i_ and [Na^+^]_i_ determinations, each successful impalement was considered one experimental *n*. We excluded all data from muscle fibers showing a resting membrane potential of less than −80 mV from the final analysis (22% of the total fibers measured). In the biochemical assay, each animal represented one experimental *N* and *n* the number of measurements. We used the D’Agostino and Pearson test to determine whether the samples were normally distributed. We compared the experimental values using a one-way analysis of variance (ANOVA) and Tukey *post hoc* test. A *p*-value <0.05 was set as statistically significant level. All statistical analyses were carried out with GraphPad Prism 9.0 (GraphPad Software, CA, United States).

## Results

### Muscle Intracellular [Ca^2+^] and [Na^+^] and Aging. Effects of FFA

**FIGURE 1:**
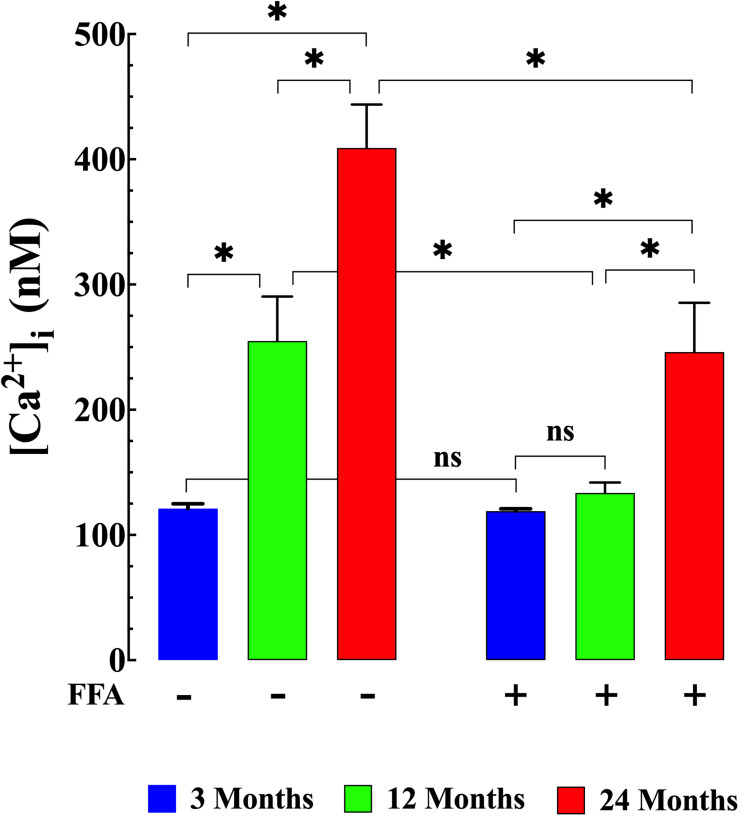
Augmented [Ca^2+^]_i_ in aged muscle fibers. Effect of flufenamic acid (FFA). Middle-aged (12-month) and aged (24-month) muscles have higher [Ca^2+^]_i_ than muscles in young animals (*p* ≤ 0.001 compared to young mice) **(left panel)**. FFA (12.5 mg/kg IP once per day for 4 weeks) normalized [Ca^2+^]_i_ in muscle cells from middle-aged animals compared to young animals (*p* > 0.65) and significantly decreased [Ca^2+^]_i_ in muscle from aged mice compared to untreated aged mice (*p* ≤ 0.001) **(right panel)**. [Ca^2+^]_i_ measurements in untreated mice were obtained from: *N* = 6 mice/age group, *n_cell_* = 14–16/age group; [Ca^2+^]_i_ measurements in FFA-treated mice were obtained from: *N* = 6 mice/per age group, *n_cell_* = 15–18/age group. Values are expressed as means ± SD. 1-Way ANOVA followed by Tukey’s *post hoc* comparisons, ^∗^*p* < 0.05.

**FIGURE 2:**
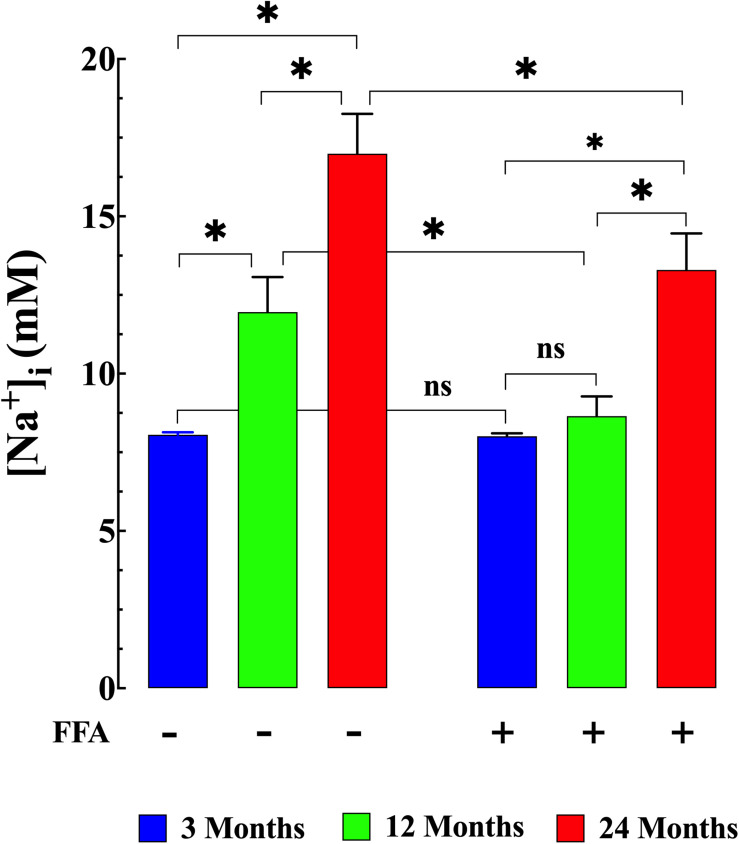
Augmented [Na^+^]_i_ in aged muscle fibers. Effect of flufenamic acid (FFA). Muscle cells from middle-aged (12-months) and aged (24-months) old mice have a higher [Na^+^]_i_ than muscle cells from young animals (*p* ≤ 0.001 compared young mice) **(left panel)**. FFA normalized [Na^+^]_i_ in middle-aged compared to young muscle (*p* ≥ 0.62) and significantly decreased in aged compared to untreated aged muscle cells (*p* ≤ 0.001) **(right panel)**. No effect of FFA treatment on [Na^+^]_i_ was observed in muscle from young mice (*p* ≥ 0.99 compared to muscle from untreated young mice). [Na^+^]_i_ measurements in untreated mice were obtained from: *N* = 6 mice/age group, *n_cell_* = 11–13/age group; [Na^+^]_i_ measurements in FFA-treated mice were obtained from: *N* = 6 mice/per age group, *n_cell_* = 11–12/age group. Values are expressed as means ± SD. 1-Way ANOVA followed by Tukey’s *post hoc* comparisons, ^∗^*p* < 0.05.

We examined muscle [Ca^2+^]_i_ and [Na^+^]_i_ *in vivo* in young, middle-aged, and aged mice to explore the changes associated with normal aging in mice. Compared to young muscle fibers [Ca^2+^]_i_ from middle-aged and aged mice was significantly elevated. In young mice, muscle [Ca^2+^]_i_ was 121 ± 4 nM (*N* = 6, *n* = 15). In middle-aged mice muscle [Ca^2+^]_i_ increased to 255 ± 36 nM (*N* = 6, *n* = 16, *p* ≤ 0.001 compared to young mice), and in aged mice it rose to 409 ± 35 nM (*N* = 6, *n* = 14, *p* ≤ 0.001 compared to young mice; Figure 1 left panel). We also found that muscle [Na^+^]_i_ is also significantly elevated in middle-aged and aged muscle compared to young mice. In muscle fibers from young mice [Na^+^]_i_ was 8 ± 0.5 mM, (*N* = 6, *n* = 11), from middle-aged mice [Na^+^]_i_ was 12 ± 1 mM (*N* = 6, *n* = 13, *p* ≤ 0.001 compared to young mice) and from aged mice [Na+]_i_ was 17 ± 1 mM (*N* = 6, *n* = 12, *p* ≤ 0.001 compared to young mice; Figure 2 left panel).

Treatment with FFA (12.5 mg/kg IP once per day for 4 weeks; see section “Materials and Methods” for more details) normalized [Ca^2+^]_i_ in muscle fibers from middle-aged mice (134 ± 8 nM, *N* = 6, *n* = 16, *p* ≥ 0.65 compared to young muscle cells) and significantly reduced [Ca^2+^]_i_ in muscle cells from aged mice (246 ± 40 nM, *N* = 5, *n* = 18, *p* ≤ 0.001 compared to untreated aged mice). No effect of FFA treatment on [Ca^2+^]_i_ was observed in muscle fibers from young mice (Figure 1 right panel). Similarly, FFA treatment normalized [Na^+^]_i_ in muscle cells from middle-aged mice (9 ± 0.7 mM. *N* = 6, *n* = 11, *p* ≥ 0.52 compared to young muscle fibers) and significantly reduced [Na^+^]_i_ in muscle from aged mice (13 ± 1 mM, *N* = 5, *n* = 12, *p* ≤ 0.001 compared to muscle from untreated aged mice). FFA did not modify [Na^+^]_i_ in young muscle cells (Figure 2 right panel).

### Increased Oxidative Stress With Aging

**FIGURE 3:**
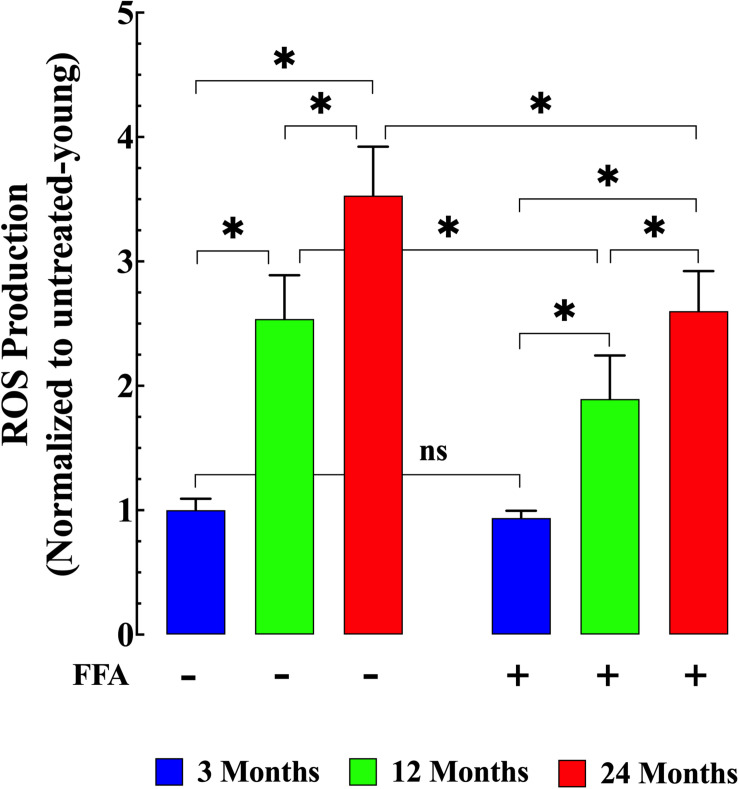
Age-related changes in skeletal muscle reactive oxygen (ROS) production. ROS production in isolated FDB fibers was significantly increased in middle-aged (*p* ≤ 0.001 compared to muscle cells from young mice) and aged fibers (*p* ≤ 0.001 compared to muscle cells from young mice) **(left panel)**. While treatment with FFA significantly reduced ROS production in middle-aged and aged muscle fibers (*p* ≤ 0.001 compared to muscle cells from untreated age-matched mice) no effect was observed in young muscle cells (*p* ≥ 0.98 compared to muscle cells from untreated young mice) **(right panel)**. ROS measurements in untreated mice were obtained from *N* = 4 mice/age group, *n_cell_* = 22–25/age group; ROS measurements in FFA-treated mice were obtained from *N* = 4 per age group, *n_cell_* = 19–25/age group. Values are expressed as means ± SD. 1-Way ANOVA followed by Tukey’s *post hoc* comparisons, ^∗^*p* < 0.05.

We measured ROS production in isolated FDB muscle cells from young, middle-aged, and aged mice. FDB muscle fibers loaded with the fluorescent ROS sensor CM-H2DCFDA in middle-aged and aged mice showed significantly increased rates of DCF fluorescence increase compared to FDB muscle fibers isolated from young mice (*p* ≤ 0.001 for both middle-aged and aged fibers compared to muscle from young mice; Figure 3 left panel). Evidence has been presented suggesting that [Ca^2+^]_i_ could modulate either ROS production and/or clearance in excitable cells (Gorlach et al., 2015). Thus, we reasoned that reducing muscle [Ca^2+^]_i_ by treatment with FFA (see Figure 1 right panel) should decrease intracellular ROS production. Pre-treatment with FFA (12.5 mg/kg IP once per day for four weeks) significantly reduced ROS production in middle-aged and aged muscle cells (*p* ≤ 0.001 compared to muscle cells from untreated middle-aged and aged mice; Figure 3 right panel). No effect of FFA treatment on ROS production was detected in muscle cells from young mice (*p* ≥ 0.98 compared to muscle cells from untreated mice; Figure 3 right panel).

### Plasma IL-6 and TNF-α Concentrations and Aging

**FIGURE 4:**
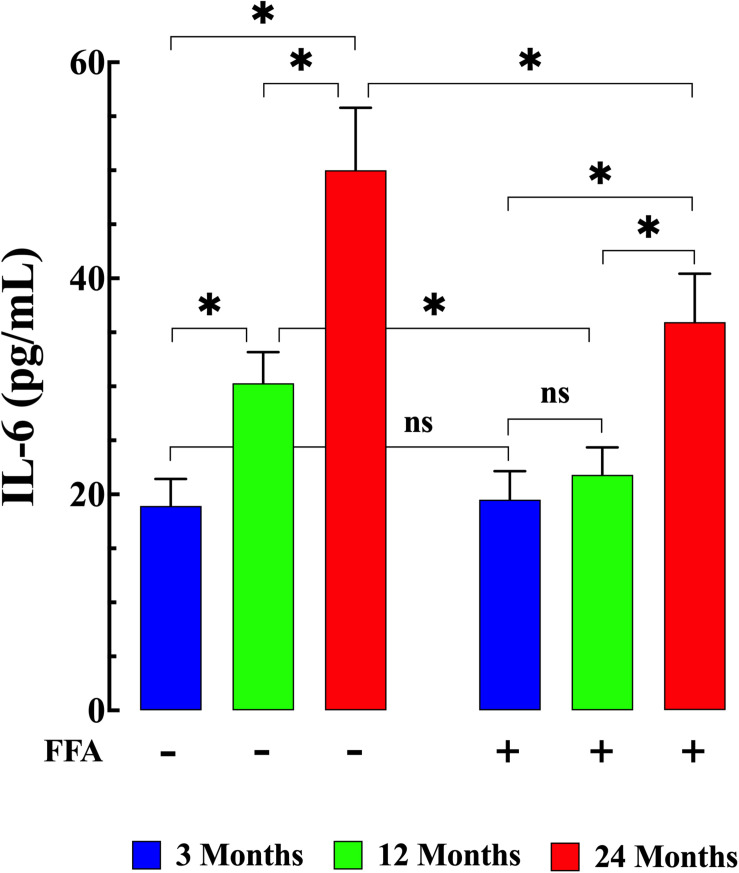
Age-associated increases in interleukin-6 (IL-6). Plasma IL6 levels were significantly increased in middle-aged and aged mice (*p* ≤ 0.001 for both) compared to plasma levels from young mice **(left panel)**. FFA treatment normalized plasma IL6 levels in middle-aged animals (*p* ≥ 0.31 compared to plasma levels from young mice), and significantly reduced plasma IL-6 in aged mice (*p* ≤ 0.001 compared to untreated aged mice). No effect on basal IL-6 levels were observed in young mice (*p* ≥ 0.99 compared to plasma levels from untreated young mice) **(right panel)**. IL-6 measurements in untreated mice were obtained from *N* = 6 mice/age group, *n* determinations = 13–16/age group; IL-6 measurements in FFA-treated mice were obtained from *N* = 6 per age group, *n* determinations = 14–16/age group. Values are expressed as means ± SD. 1-Way ANOVA followed by Tukey’s *post hoc* comparisons, ^∗^*p* < 0.05.

**FIGURE 5:**
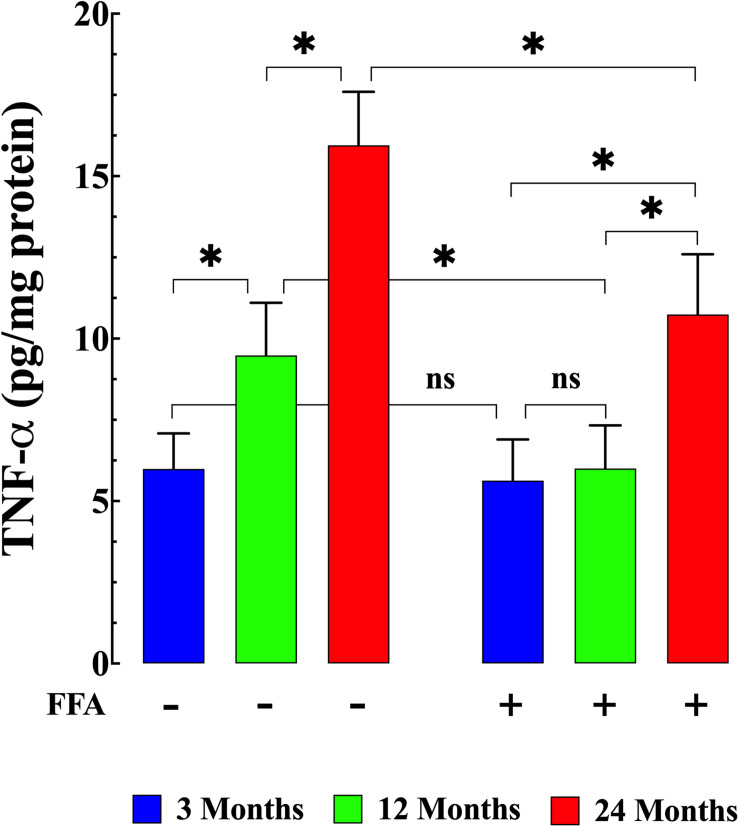
Increased TNF-α plasma level and aging. Middle-aged and aged mice had significantly TNF-α higher levels than young mice (*p* ≤ 0.001) **(left panel)**. FFA treatment normalized plasma TNF-α in middle-aged animals (*p* ≥ 0.99 compared to plasma levels from young mice), and although it did not normalize plasma TNF-α in aged mice, it significantly reduced plasma TNF-α levels compared to untreated aged mice **(right panel)** (*p* ≤ 0.001). FFA treatment had no effect on basal TNF-a levels in young animals (*p* ≥ 0.98 compared to untreated young mice) **(right panel)**. TNF-α measurements in untreated mice were obtained from *N* = 6 mice/age group, *n* determinations = 15–18/age group; TNF-α measurements in FFA-treated mice were obtained from *N* = 6 per age group, *n* determinations = 16–18/age group. Values are expressed as means ± SD. 1-Way ANOVA followed by Tukey’s *post hoc* comparisons, ^∗^*p* < 0.05.

Senescence has been shown to be associated with an increase in inflammatory markers such as IL6 and TNF-α (Wei et al., 1992; Garner et al., 2018). Therefore, we measured IL-6 levels and TNF-α levels in plasma from young, middle-aged, and aged mice. We found that plasma IL-6 concentration increased from 19 ± 2 pg/mL (*N* = 6 mice, *n* = 13) in young mice, to 30 ± 3 pg/mL in middle-aged mice (*N* = 6 mice, *n* = 14, *p* ≤ 0.001 compared to young mice), and to 50 ± 6 pg/mL in aged mice (*N* = 6 mice, *n* = 16, *p* ≤ 0.001 compared to young mice), (Figure 4 left panel). Similarly, we found that there was an age-dependent elevation of plasma TNF-α concentration from 5.9 ± 1.1 pg/mg^–1^ protein (*N* = 6 mice, *n* = 15) in young mice to 9.6 ± 1.6 pg/mg protein middle-aged mice (*N* = 6 mice, *n* = 16, *p* ≤ 0.001 compared to young mice) and to 15.9 ± 2.1 pg/mg protein in aged mice (*N* = 6 mice, *n* = 18, *p* ≤ 0.001 compared to young mice; Figure 5 left panel). Treatment with FFA (12.5 mg/kg IP once per day for four weeks) normalized plasma IL-6 levels in middle-aged mice (22 ± 3 pg/mL, *N* = 6 mice, *n* = 16, *p* ≥ 0.31 compared to plasma levels from young mice and *p* ≤ 0.001 compared to plasma IL-6 levels from untreated middle-aged mice). In aged mice FFA reduced plasma IL-6 levels to 36 ± 4 pg/mL (*N* = 6 mice, *n* = 15, *p* ≤ 0.001 compared to plasma IL-6 levels from untreated aged mice; Figure 4 right panel). Likewise FFA treatment significantly reduced TNF-α levels in both middle-aged mice group where it was normalized to the levels of untreated young mice (6 ± 1.3 pg/mg protein, *N* = 6 mice, *n* = 15, *p* ≥ 0.99 compared to plasma levels from young mice) and in the aged group it was reduced to 10.7 ± 1.9 pg/mg protein, *N* = 6 mice, *n* = 18, *p* ≤ 0.001 compared to plasma concentrations from untreated aged-mice; Figure 5 right panel) In young mice, FFA did not change the IL-6 plasma level (20 ± 3 pg/mL, *N* = 6 mice, *n* = 14, *p* ≥ 0.99 compared to plasma IL-6 levels from untreated young mice; Figure 4 right panel) or TNF-α levels (5.6 ± 1.2 pg/mg protein, *N* = 6 mice, *n* = 16, *p* ≥ 0.98 compared to plasma concentration from untreated aged-mice; Figure 5 right panel).

### Muscle Damage and Aging

**FIGURE 6:**
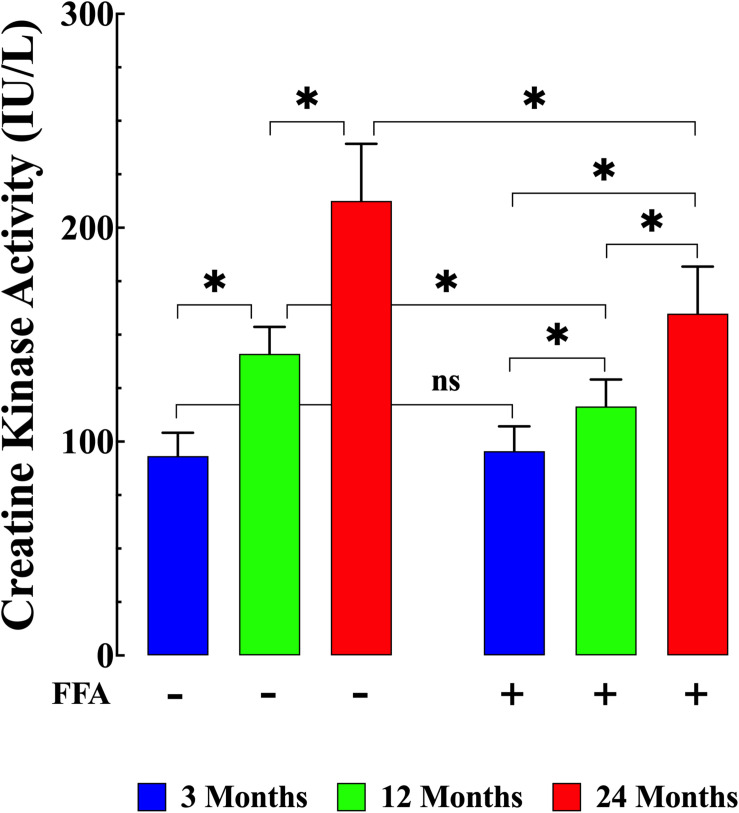
Variation of creatine kinase (CK) levels with aging. CK levels were significantly higher in middle-aged and aged mice (*p* ≤ 0.001) compared to plasma levels young mice **(left panel)**. FFA treatment normalized plasma CK levels in middle-aged animals compared to young animals (*p* ≥ 0.99 compared to plasma levels from young mice) and FFA significantly reduced plasma CK concentrations in aged mice compared to untreated aged mice (*p* ≤ 0.001). FFA did not modify basal CK levels in young animals (*p* ≥ 0.99 compared to plasma levels from untreated young mice). CK measurements in untreated mice were obtained from *N* = 6 mice/age group, *n* determinations = 19–20/age group; CK measurements in FFA-treated mice were obtained from *N* = 6 per age group, *n* determinations = 16–17/age group. Values are expressed as means ± SD. 1-Way ANOVA followed by Tukey’s *post hoc* comparisons, ^∗^*p* < 0.05.

Although an increase in plasma CK can indicate acute cardiac muscle damage, levels which remain increased over time are more likely to be an indication of skeletal muscle damage which has previously been shown to be associated with aging (Kim et al., 2018). Here we found that plasma CK activity was significantly elevated in both middle-aged mice (141 ± 13 IU/L, *N* = 6 mice, *n* = 18) and aged mice (213 ± 27 IU/L, *N* = 6, *n* = 20) compared to young mice (95 ± 11 IU/L *N* = 6 mice, *n* = 20 *p* ≤ 0.001 compared to middle-aged and aged mice; Figure 6 left panel). FFA treatment significantly lowered CK levels in middle-aged (117 ± 13 IU/L *N* = 6, *n* = 17, *p* ≤ 0.001 compared to untreated middle aged-mice) and in aged mice (162 ± 23 IU/L *N* = 6, *n* = 18, *p* ≤ 0.001 compared to untreated aged-mice; Figure 6 right panel). As seen with the increase in inflammatory markers, FFA had no effect on plasma CK levels in young animals (96 ± 12 IU/L (*N* = 6, *n* = 16, *p* ≥ 0.99 compared to untreated mice; Figure 6 right panel).

## Discussion

In this study we directly examined changes in resting intracellular Ca^2+^ and Na^+^ homeostasis in skeletal muscle during normal aging. The major findings of the present study are an age-dependent increase of skeletal muscle [Ca^2+^]_i_, [Na^+^]_i_, increased ROS generation and elevation of plasma IL-6, TNF-α and CK concentrations (aged > middle-aged > young). Treatment with FFA, reduced muscle levels of [Ca^2+^]_i_, [Na^+^]_i_, reduced ROS production and reduced plasma levels of IL-6, TNF-α and CK in middle aged and aged mice (12 and 24-months) but had no effect in young mice (3-months).

Regulation of resting intracellular calcium [Ca^2+^] is critical in muscle cells. While extracellular Ca^2+^ concentration ranges within 1–2 mM in quiescent and healthy muscle cells resting free cytosolic [Ca^2+^] is remarkably low (10^–7^ M) (Marban et al., 1980; Lopez et al., 1983, 2018a). Several intracellular regulatory mechanisms have been proposed in muscle cells to preserve this low resting [Ca^2+^]_i_. ATP-driven Ca^2+^ pumps pump out Ca^2+^ from the cytoplasm, including the sarcoplasmic reticulum (SR) Ca^2+^ATPase that pumps it back into the SR and the plasma membrane Ca^2+^-ATPase (Brini and Carafoli, 2009) that pumps it out of the cytoplasm into the extracellular space. Furthermore [Ca^2+^]_i_ is also regulated by Ca^2+^ influx through TRPC channels (Choi et al., 2020), store-operated Ca^2+^ channels (Lyfenko and Dirksen, 2008), the bidirectional and electrogenic Na^+^/Ca^2+^ exchanger in the plasma membrane (Cifuentes et al., 2000; Fraysse et al., 2001; Blaustein, 2013) and by passive leak from the SR through the ryanodine receptor (Yang et al., 2007; Eltit et al., 2010; Andersson et al., 2011; Lamboley et al., 2016).

Transient receptor potential canonical channels are non-selective cation channels localized in mammalian cells’ plasma membrane. Among the TRPCs, in skeletal muscle, TRPC3 is the most expressed, followed by TRPC1 and TRPC6 (Kunert-Keil et al., 2006). TRPC channels have been implicated in the pathogenesis of diverse muscle diseases, in particular muscle dystrophies and myasthenia gravis (Kruger et al., 2008; Gailly, 2012; Mijares et al., 2014; Sauc and Frieden, 2017; Lopez et al., 2020). Furthermore, overexpression of a dominant-negative form of TRPC6 (dnTRPC6) in this TRPC3 overexpression background reverted its dystrophic phenotype as well as reverting the dystrophic phenotype both in mdx and in Scgd mouse (Millay et al., 2009; Burr et al., 2014).

Our data show that muscle fibers from aged mice have an elevated resting intracellular [Ca^2+^]_i_ and [Na^+^]_i_ compared with young muscle cells. The elevated resting [Ca^2+^]_i_ and [Na^+^]_i_ found in aged muscle cells is consistent with our recent report showing that [Ca^2+^]_i_ is increased in aged neurons compared to young neurons (Uryash et al., 2020). The failure to maintain a low resting [Ca^2+^]_i_ in muscle cells can activate enzymes such as proteases, nucleases, and lipases that impair energy production, compromise muscle function and provokes cell death (Nicotera and Orrenius, 1998; Altamirano et al., 2014a). The underlying mechanisms related to the observed dysfunction of [Ca^2+^]_i_ in aging muscle is complicated. However, an increase in RyR1 leak has been reported in aged muscle (Andersson et al., 2011; Lamboley et al., 2016). This leak then provokes a chronic partial depletion of SR Ca^2+^ which induces an increased influx of extracellular Ca^2+^ that is independent of the L-type Ca^2+^ channel mediated Ca^2+^ entry (Launikonis and Rios, 2007; Lyfenko and Dirksen, 2008). This pathway’s identity is not fully understood; however, the involvement of TRPCs, which are highly expressed in skeletal muscle cells, has been suggested (Vandebrouck et al., 2002; Millay et al., 2009). A similar aberrant elevation in [Ca^2+^]_i_ and [Na^+^]_i_ have been observed in muscle cells from patients with Duchene’s muscular dystrophy and mdx mice (Lopez et al., 1987; Altamirano et al., 2014b; Lopez et al., 2017, 2020) where an abnormal Ca^2+^ influx has also been reported (Tutdibi et al., 1999; Kruger et al., 2008; Millay et al., 2009). Thus, if it were possible to prevent this chronic elevation of [Ca^2+^]_i_ during aging, it may exert a myo-protective effect and potentially prevent the muscle wasting and dysfunction observed in the aging muscle.

We show here that there is a significant increase in ROS generation in aging muscles (old > middle-aged > young). Physiological concentrations of ROS play essential roles in diverse muscle signaling pathways. However, elevated ROS production harms muscle function (Wanagat et al., 2001; Calvani et al., 2013; Le Moal et al., 2017). ROS and lipid peroxidation levels are abnormal in senescent muscle cells (Ryan et al., 2010). However, there is a large body of evidence showing mitochondrial dysfunction is associated with aging, and it is well known that the mitochondria are a primary source of cells ROS production (Boengler et al., 2017). Intracellular ROS dyshomeostasis has been suggested to decrease protein synthesis and increase protein degradation, provoking muscle atrophy (Kinugawa et al., 2015) and microdamage of the muscle membrane allowing release of intracellular components such as CK (Kim et al., 2018). One of the results of increased ROS production is an increase in the plasma levels inflammatory markers such as IL-6 and TNF-α such as we found here. The above-described results suggest that increased ROS production in senescent skeletal muscle might be linked with age-dependent intracellular Ca^2+^ imbalance observed in aging mice (Kinugawa et al., 2015).

To further explore the mechanism involved in the changes of [Ca^2+^]_i_, [Na^+^]_i_, ROS, IL-6, TNF-α with aging we treated mice with FFA, a NSAID drug which also blocks TRPCs which are non-selective plasmalemmal cation channels (Chen et al., 1993; Hescheler and Schultz, 1993). The FFA anti-inflammatory effect appears to be mediated by reduction of prostaglandin synthesis by inhibiting the cyclo-oxygenases (Flower, 1974). Clinically FFA has been used locally for analgesia against pain and inflammation associated with musculoskeletal and joint disorders, peri-articular, and soft tissue disorders (Flower, 1974). In addition, FFA has been used as a non-selective plasmalemmal cation entry channel blocker and has been shown to inhibit the spontaneous active tone of carotid artery (Shimamura et al., 2002) and to attenuate the K^+^-induced contraction in endothelium-denuded small and large arteries (Bencze et al., 2015). In this study, we demonstrated that administration of FFA reduced intracellular Ca^2+^ and Na^+^ overload, decreased the rate of ROS production, and lowered the high plasma concentration levels of IL-6 and TNF-α and CK activity in aging mice. Due to FFA’s lack of pharmacological specificity (anti-inflammatory and TRPC channel blocker), we are unable to dissect which of these is the primary mechanism of action and which is the result of the primary action.

## Conclusion

In this study we present direct evidence of abnormal regulation of [Ca^2+^]_i_, [Na^+^]_i_, and increased ROS production in aging muscles. Furthermore, we show that aging is associated with elevated plasma levels of the inflammatory markers, IL6, TNF-α, and CK which is an indicator of chronic muscle damage. Treatment with FFA significantly decreased elevated [Ca^2+^]_i_, [Na^+^]_I_, and reduced ROS overload, which was accompanied by decreases in plasma IL6, TNF-α, and CK levels. The mechanism of FFA’s action in correcting these age related defects may be related either to its action as a TRPC channel blocker and/or its direct anti-inflammatory effect on ROS production.

## Data Availability Statement

The raw data supporting the conclusions of this article will be made available by the authors, without undue reservation.

## Ethics Statement

All protocols used in the study were performed following the recommendations in the Guide for the Care and Use of Laboratory Animals of the National Institutes of Health and approved by the IACUC of the Mount Sinai Medical Center, United States.

## Author Contributions

AM and JL performed the research and analyzed the data. AM, JL, and PA wrote the manuscript. All authors contributed to the manuscript revision and read and approved the submitted version.

## Conflict of Interest

The authors declare that the research was conducted in the absence of any commercial or financial relationships that could be construed as a potential conflict of interest.
